# Macauba (*Acrocomia aculeata*) as a Bioenergy Platform: Integrated Biodiesel Production and Lignocellulosic Biomass Valorization

**DOI:** 10.1021/acsomega.5c10973

**Published:** 2025-12-12

**Authors:** Daniel Chagas Nascimento, Ewerton Henrique de Souza Santos, Fábio Moreira da Silva, Diego Coelho Barroso dos Santos, Edgar Amaral Silveira, Janaína Heberle Bortoluzzi, Grace Ferreira Ghesti, Mario Roberto Meneghetti, Paulo Anselmo Ziani Suarez, Simoni Margareti Plentz Meneghetti

**Affiliations:** † Laboratory of Bioprocess, Materials and Fuels − Institute of Chemistry, 28112 University of Brasília, P.O. Box 4478, Brasília, DF 70919-970, Brazil; ‡ Group of Catalysis and Chemical Reactivity− Institute of Chemistry and Biotechnology, Federal University of Alagoas, Maceió, AL 57072-000, Brasil; § Mechanical Sciences Graduate Program, Laboratory of Energy and Environment, 28127 University of Brasília, Brasília, DF 70919-970, Brazil

## Abstract

Macauba (*Acrocomia aculeata*) is a native palm from tropical regions with high potential for integrated bioenergy and bioproduct applications. This study evaluated the physicochemical properties of macauba pulp and kernel oils, assessed the production and fuel properties of biodiesel derived from commercial pulp oil, and explored the use of macauba lignocellulosic biomass for biochar production. Pulp oil exhibited a high oleic acid content, which makes it highly suitable for biodiesel synthesis since oleic acid forms esters with good oxidative stability, adequate fluidity, and high combustion quality, resulting in a biodiesel that readily meets fuel standards. Biodiesel was obtained through a two-step process, and its blends with fossil diesel (DS10 and DS500: commercially available fossil diesel fuels containing up to 10 mg kg^–1^ and 500 mg kg^–1^ of sulfur, respectively) were evaluated in terms of density, kinematic viscosity, and heating value. The results indicate that up to 60% (v/v) methylmacauba biodiesel (MMB) can be blended with DS10 and up to 90% with DS500 without exceeding regulatory limits. Additionally, biodiesel production in a continuous tubular reactor confirmed its technical feasibility, yielding more than 70% under optimized conditions. Pyrolysis of macauba biomass at 400–600 °C generated biochar with increased carbon content, energy density, and structural ordering. Biochars derived from extractive-containing biomass exhibited distinct elemental and energetic profiles compared with extractive-free samples. These findings reinforce the suitability of macauba as a versatile and sustainable feedstock for biorefinery platforms.

## Graphical abstract

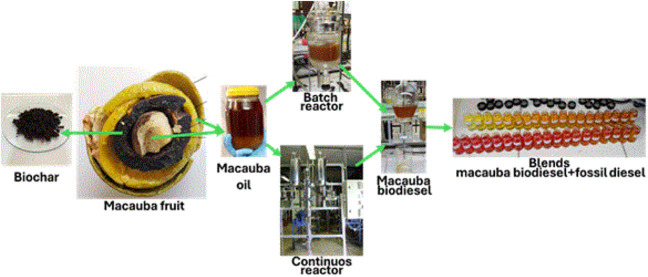

## 1 Introduction

The increasing global demand for sustainable energy sources has intensified the search for renewable feedstocks capable of replacing fossil fuels.^1^ Regarding the substitution of fossil diesel, several countries, such as Brazil, the United States, European countries, and Malaysia, have launched biodiesel programs using soybean, canola, and the palm tree *Elaeis guineensis* edible oils. However, the use of biodiesel has become controversial because of a trilemma: balancing food, energy, and environment.^2^ Thus, the production of biodiesel in Brazil is nowadays using up to 25% of alternative and cheaper fatty acid raw materials from different sources, such as acid stocks produced during physical neutralization of fats and oils; domestic or industrial sludge; and poultry, porcine, or cattle slaughterhouse wastes. In this context, native plants from tropical regions, especially when they are highly productive even when grown in consort with cattle production or another crop plantation, offer a valuable opportunity for the development of integrated bioenergy and food production systems.^3,4^

Among these, *Acrocomia aculeata*, commonly known as Macauba, stands out due to its high productivity, adaptability to different ecosystems, and the energetic potential of its oil and lignocellulosic biomass.^5^ Macauba is a palm tree widely distributed in South America, especially in Brazil, where it occurs naturally in the Cerrado, Caatinga, and Atlantic Forest biomes. It is worth mentioning that it grows naturally in cattle farms in the Brazilian Savannah (Cerrado biome) and is commonly used by farmers for animal thermal comfort without compromising grass supply. Its fruit is composed of a hard endocarp, an oil-rich fleshy pulp, and an oil-rich kernel. Thus, both the pulp and kernel contain significant amounts of lipids, making macauba one of the most promising oleaginous species for biodiesel production in tropical regions. In addition to the oil fractions, the shell and endocarp represent abundant lignocellulosic residues with high fixed carbon content, which can be thermochemically converted into charcoal or biochar with potential applications in energy generation and soil amendment.^6,7^

Several studies have reported the use of macauba oil in transesterification processes for biodiesel production, highlighting its favorable fatty acid profile and high yield per hectare.^8–13^

High oleic acid content makes macauba oil highly suitable for biodiesel synthesis because oleic acid (C18:1) produces methyl esters with an optimal balance of oxidative stability, cold-flow properties, and cetane number. Oils rich in oleic acid generate biodiesel with low susceptibility to oxidation (unlike polyunsaturated fatty acids) and good fluidity at moderate temperatures (better than highly saturated fats). In addition, oleic-based biodiesel exhibits a high combustion quality, contributing to efficient engine performance.

Simultaneously, research on the pyrolysis of macauba residues has demonstrated the feasibility of obtaining carbonaceous materials with suitable calorific value and porous structure for thermal applications.^6,7^ The integration of these energy routes—liquid biofuels and solid bioenergy products—strengthens the role of macauba as a strategic species for the development of sustainable biorefineries.

This study aims to explore the energetic potential of macauba through the characterization and valorization of its main parts. Emphasis is given to the use of pulp oil for biofuel production as well as the conversion of shell-derived biomass into charcoal and biochar, contributing to an integrated and sustainable approach for tropical biomass utilization. Although macauba has been studied as a source of oil or lignocellulosic residues, these routes are usually evaluated separately. The novelty of this study lies in providing an integrated assessment of the energetic potential of the pulp oil and shell biomass within the same framework, demonstrating how both fractions can be simultaneously valorized for biodiesel and solid biofuels. This combined approach fills a gap in the literature and advances the concept of a unified macauba-based biorefinery.

## 2 Methodology

### 2.1 Materials

Analytical grade H_2_SO_4_, *n*-hexane, and CH_3_OH (MeOH) were purchased from Merck (Darmstadt, Germany). Reagent grade NaOH, NaHCO_3_, NaCl, MgSO_4_, and H_3_PO_4_ were obtained from Vetec (São Paulo, Brazil). All of the reagents were used without further purification.

### 2.2 Collection of Macauba Fruits and Separation of Pulp and Kernel

A total of 200 macauba fruits were collected from a 4-m-tall palm bearing five bunches, located in the Parque Ecológico de Águas Claras, Brasília, Federal District, Brazil (15.8360° S, 48.0252° W). After harvesting, the fruits were manually peeled and depulped by using a hammer and pruning pliers. The endocarp was cracked open to separate the kernel. Both the pulp and kernel were stored in a freezer to prevent deterioration. Subsequently, the materials were dried at 60 °C for 24 h and then ground.

### 2.3 Extraction and Characterization of Macauba Pulp and Kernel Oils

The oil was extracted from the ground materials using a Soxhlet apparatus with *n*-hexane, following the official AOAC method 963.15 (A.O.A.C., 1976). The oil content was also determined by this method. The fatty acid composition of the oils was analyzed by gas chromatography (GC) according to AOCS Official Methods Ce 1-62 and Ce 2-26. The acid value of the samples (expressed in mg KOH·g^–1^ oil) was determined using an automatic titrator (Metrohm 907 Titrando), following AOCS Official Method Cd 3d-63. The dynamic viscosity and density of the liquid samples were determined using a rotational Stabinger viscodensimeter (Anton Paar SVM 3000), which operates based on the modified Couette principle with a rapidly rotating outer tube. Prior to measurements, the instrument was calibrated according to the manufacturer’s specifications using certified reference standards for both viscosity and density. Approximately 3–5 mL of each sample was injected into the measurement cell, and the analyses were performed under controlled temperature conditions, following ASTM D7042.

### 2.4 Characterization of Commercial Unrefined Macauba Oil

The commercial unrefined macauba pulp oil was obtained from the Cooperriachão cooperative, located in Mirabela, Minas Gerais, Brazil. The oil was extracted through cold mechanical pressing followed by filtration to remove solid particles. It was stored in sealed containers and kept in a freezer at −15 °C. The fatty acid composition of this oil was determined by gas chromatography (GC), using AOCS Official Methods Ce 1-62 and Ce 2-26, and the acid value (mg KOH g^–1^ oil) was determined using the Metrohm 907 Titrando automatic titrator, following AOCS Official Method Cd 3d-63.

### 2.5 Production of Biodiesel from Commercial Unrefined Macauba Oil: Methylic Macauba Biodiesel (MMB)

Due to the high acidity of the commercial Macauba oil, the biodiesel production process involved two stages: fatty acid esterification followed by triacylglycerol transesterification. The esterification reaction was performed using macauba pulp oil with MeOH as an alcoholysis agent and sulfuric acid (H_2_SO_4_) as the catalyst. A molar ratio of 1:8 (oil:MeOH) and 1 wt % catalyst relative to the oil mass were used, with a reaction time of 2 h at 60 °C. The reactions were carried out in a jacketed glass reactor equipped with a condenser and heating system (Tecnal, model TEC-BIO; 2 L total volume, with a height of 30 cm and a diameter of 18 cm). The system includes a 304 stainless-steel lid equipped with ports for sensors, the stirrer shaft, chemical dosing lines, an air inlet, a septum, and a sampling system. Stainless-steel rings and rods ensured structural support and effective sealing, while unused openings were closed with sealing nipples. The reactor also contained a four-blade flat baffle ring, autoclavable silicone hoses, and a stainless-steel support frame. Mechanical agitation was performed using a Tecnal mechanical stirrer (model TE-139, Brazil), equipped with a stainless-steel shaft and a naval-type impeller. The stirrer operates with analog speed control (1500 rpm in this work), a brushed DC motor, and a nominal power of 150 W at 220 V. After the reaction, the esterified oil was transferred to a 2.0 L separation funnel and left to rest for phase separation. Since the pH of the esterified oil was approximately 1, it was neutralized with a 5 wt % sodium bicarbonate solution until reaching a pH close to 7. The oil was also washed with a sodium chloride (NaCl) solution. After phase separation, the oil was stored in a glass container with a desiccant agent (magnesium sulfate). The transesterification reaction was carried out using the esterified oil, methanol (MeOH), and sodium hydroxide (NaOH) as the catalyst. A molar ratio of 1.0:9.0:0.2 (oil:alcohol:catalyst) was utilized, with a reaction time of 2 h at 60 °C. The same reactor used for esterification was employed. After the reaction, the mixture was transferred to a 2.0 L separation funnel and left to rest for 3 h to allow complete separation of the reaction products, biodiesel and glycerol. The obtained biodiesel was neutralized with a 5 vol % phosphoric acid solution and washed with sodium chloride (NaCl) solution until a pH of 7.0 was achieved. For each washing step, approximately 200 mL of NaCl solution was added to the separation funnel, the mixture was shaken, and the washing solution was discarded. This process was repeated until complete neutralization. The resulting MMB was stored in a refrigerator for subsequent physicochemical analyses. The biodiesel yield was determined by gas chromatography.^14^ Analyses were performed using a Shimadzu GCPlus chromatograph equipped with a flame ionization detector and a 2.2 m column, with an injector temperature at 250 °C, detector temperature at 340 °C, column temperature at 50 °C, and column pressure at 6 kPa. Approximately 0.15 g of sample was dissolved in 1 mL of a solution prepared with 10 mL hexane and 0.08 g of glycerol trioctanoate (tricaprylin). An injection volume of 1 μL was used, with a total chromatographic run time of 20 min, using hydrogen as the carrier gas.

### 2.6 Acquisition of S10 and S500 Diesel

Commercially available fossil diesel samples (S10 and S500, indicating sulfur contents of up to 10 mg kg^–1^ and 500 mg kg^–1^, respectively), free from biodiesel (its incorporation is mandatory for commercialization in the Brazilian market), were obtained from a fuel distribution company. In this study, they were referred to as DS10 and DS500.

### 2.7 Preparation of Macauba Biodiesel and Diesel Blends and Determination of Physicochemical Parameters

Following the production of MMB, blends were prepared by using varying biodiesel volumes (from 5% to 95%) with both types of commercial diesel (DS10 and DS500). Physicochemical analyses were then performed on both pure samples and blends. Kinematic viscosity was determined according to ASTM D445-12, using a capillary viscometer in which 8.5 mL of sample was placed, and the time required for the liquid to flow between the upper and lower menisci was recorded. Measurements were conducted in triplicate at a thermostatic bath temperature of 40 °C. Density, or specific mass, was determined according to ASTM D4052-11 at 20 °C, also in triplicate, by using a digital densimeter. The heating value was determined in triplicate using an IKA-C200 bomb calorimeter following ASTM D-2382 and ABNT NBR 8633/84 standards.

### 2.8 Synthesis and Preparation of Catalysts for Use in the Continuous Tubular Reactor

Catalysts of mixed aluminum and zinc oxides (alumina doped with zinc (Al_2_O_3_)_4_(ZnO)) and aluminum and tin mixed oxides (alumina doped with tin (Al_2_O_3_)_4_(SnO))^15,16^ were synthesized following the method by Macedo et al.^17^ This involved coprecipitation of the precursor in an alkaline medium, followed by purification and calcination in air to obtain the mixed oxide. The powdered catalysts (particle size <250 μm) were shaped into pellets for use inside the continuous tubular reactor. Cylindrical pellets, 2 mm in diameter and 1 cm in length, were prepared by extruding a catalyst paste using a Bonnot Co. “BB Gun” extruder with a 2 mm mold, following the methodology described by Silva et al.^16^ The extruded paste composition for each catalyst is shown in Table 1.

**Table 1:** Mass Composition of the Extruded Paste for the Catalyst Pellet Preparation

Formulation	Catalyst	Catalyst (g)	Starch (g)	Water (g)
1	(Al_2_O_3_)_4_(ZnO)	100,0	2,5	48,0
2	(Al_2_O_3_)_4_(SnO)	100,0	2,5	58,4

After extrusion, the pellets were dried in an oven at 110 °C to a constant weight. Calcination at 500 °C for 4 h in a tubular furnace (with low air flow and a heating rate of 10 °C min^–1^) was performed to remove the starch. The useful volume of each catalyst bed (pellets) was determined and loaded into a continuous tubular reactor for catalytic activity evaluation. The specific surface area of the calcined pellets, obtained by N_2_ adsorption–desorption analysis (Quantachrome Nova 2000e), was approximately 4.7 m^2^ g^–1^. Before analysis, the sample was pretreated at 150 °C for 3 h under a vacuum to remove moisture and surface-adsorbed species. The average pore diameter was 27.17 Å.

### 2.9 Evaluation of Biodiesel Production from Macauba Oil in a Continuous Tubular Reactor

The continuous pilot system with a tubular reactor used for the reactions is shown in Figure 1.^15,16^ The MeOH and oil flow rates from the storage tanks (1) were controlled by automatic flow regulators (2), followed by mixing in a static mixer (3), and then contacting the catalyst pellets inside the continuous tubular reactor (4) with heating control and a total volume of 4.5 L. The reaction product was cooled in a shell-and-tube heat exchanger (5), and a manual needle valve (6) regulated the product outflow under reaction pressure and temperature. The storage tanks were pressurized with nitrogen gas, as the system lacked pumps for displacing the reagents through the catalyst bed.

**Figure 1:**
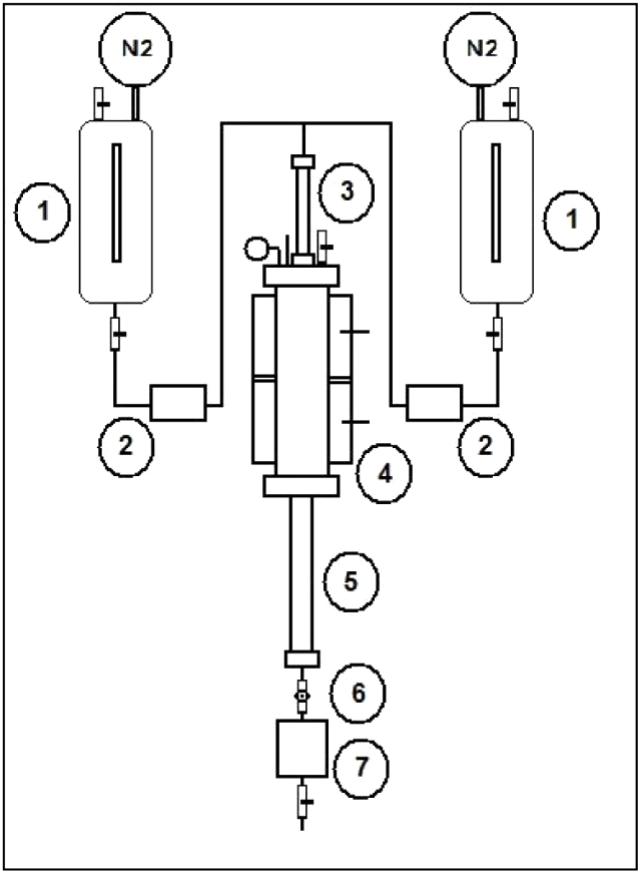
Continuous pilot system with tubular reactor: (1) methanol and/or oil tank, (2) methanol and/or oil flow regulator, (3) static mixer, (4) tubular reactor with heating jacket, (5) shell-and-tube heat exchanger, (6) needle valve, and (7) product collection tank.

Catalytic activity tests for each catalyst bed were conducted in the 4.5 L continuous tubular reactor using commercial unrefined macauba oil under varying temperature conditions (140 °C and 150 °C), MeOH-to-oil molar ratios (6 and 9), and reactor residence times (4 h and 6 h), as shown in Table 2. The reactor residence time was counted from the moment the reactor reached the specified temperature and flow conditions. The maximum temperature of 150 °C was set based on the operating limits of the tubular reactor. These conditions were selected based on previous studies.^15,16^

**Table 2:** Sequence and Conditions of Transesterification Reactions in the Continuous Prototype for Each Catalyst Bed^a^

Entry	T_reactor_ (°C)	Molar ratio MeOH:oil	Residence time (h)	Total reaction time (h)
1	140	6	4	8
2	140	6	6	12
3	140	9	4	8
4	140	9	6	12
5	150	6	4	8
6	150	6	6	12
7	150	9	4	8

a T_reator_ = Reactor temperature.

The yield analysis methodology combined a titrimetric acid index determination using a methanolic KOH solution and high-performance liquid chromatography (HPLC), following the approach proposed by Carvalho et al. (2012).^13^ HPLC analyses were conducted using a Shimadzu CTO-20A liquid chromatograph with UV detection at 205 nm, employing a Shim-Pack VP-ODS (C-18, 250 mm, 4.6 mm i.d.) column maintained at 40 °C.

### 2.10 Preparation of Macauba Biochar

In this thermoconversion process, approximately 3 g of biomass (macauba endocarp) were weighed into porcelain crucibles and placed in a tubular furnace where pyrolysis was performed at varying temperatures (400 °C, 500 °C, and 600 °C), while keeping the other parameters constant (30 °C min^–1^ heating rate and residence time of 60 min). The yield (wt %) was determined gravimetrically, and the biochar was characterized using several analytical techniques. The elemental composition of carbon (C), hydrogen (H), and nitrogen (N) was determined using a LECO CHN628 elemental analyzer, and approximately 2.0 mg of dried and finely ground sample was accurately weighed into a tin capsule and introduced into the combustion chamber of the instrument. The sample was completely oxidized in an oxygen-rich atmosphere at a temperature of approximately 950 °C. The oxygen content was calculated by the difference (O (%) = 100% – (C% + H% + N%)). The HHV of the samples was calculated using eq 4 (Ghesti et al., 2022)^30^.

1 HHV=−1.3675+(0.3137×C)+(0.7009×H)+(0.0318×O)

For thermogravimetric analysis (TGA), approximately 5 mg of each sample was subjected to continuous heating to 800 °C under a constant nitrogen flow of 100 mL min^–1^, using a TA Instruments SDT 2960 system. The resulting data were processed in Excel, and DTG (mass derivative with respect to temperature) versus temperature (°C) plots were generated using Origin software. In the case of Fourier-Transform Infrared Spectroscopy (FTIR), KBr pellets were prepared using a 150:1 (wt:wt) ratio of KBr to sample, and analyses were carried out using a Shimadzu IRAffinity-1 spectrometer. For physisorption analysis (BET), approximately 1 g of sample was used. The samples were first degassed in the analysis port of a NOVA 2200e instrument (Quantachrome) at 200 °C for 4 h. After drying, nitrogen gas was used for adsorption measurements. Raman analyses were conducted using a Renishaw InVia Raman spectrometer equipped with a 632.8 nm HeNe laser (maximum power 20 mW). The effective power at the laser output was set to 16.4 mW. The graphitization index of the biochars was determined from the Raman spectra as the intensity ratio of the D-band (related to structural disorder) to the G-band (associated with C=C bond stretching).

## 3 Results and Discussion

### 3.1 Macauba Fruit

As already mentioned, Macauba (*Acrocomia aculeata*) fruit contains a hard lignocellulosic endocarp, an oil-rich fleshy pulp, and a kernel composed of an oil-rich nut recovered by a hard lignocellulosic tegument (Figure 2). For samples prepared with dried ripe fruits (endocarp, pulp, and kernel), the oil yield, defined as the mass of oil extracted from the total sample mass, averaged 18%. The oil content of the samples on a dry basis was 38% for pulp and 51% for kernels.

**Figure 2:**
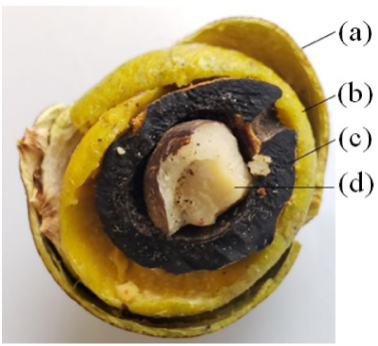
Macauba fruit and its components: (a) outer shell, (b) pulp, (c) endocarp, (d) kernel.

While both parties can offer high oil content, their compositions and industrial applications can differ significantly, as depicted in Table 3. The oil extracted from Macauba kernel is compositionally like palm tree *Elaeis guineensis* kernel oil, characterized by a high proportion of medium-chain saturated fatty acids. This profile makes Macauba kernel oil particularly attractive to the cosmetic and personal care industries, where it is used in the formulation of soaps, creams, and emollients. On the other hand, Macauba pulp oil has a large amount of long-chain monounsaturated fatty acids, with its composition like canola and olive oils. Thus, it is reasonable to think that Macauba pulp oil would be especially attractive to the biofuel industry. It is important to highlight that the composition of Macauba pulp and kernel oil available in the literature is in good agreement with the values found in this work, as may be compared from Table 3.^10,18,19^ The acidity index was low, with values of 3.77% for the pulp oil and 1.50% for the kernel oil.

**Table 3:** Pulp and Kernel Macauba Oils Composition

		This work			Literature^10,18,19^	
		Pulp	Kernel	Comercial^a^	Pulp	Kernel
Caproic acid	C6:0	-	0.3	-	-	0.8
Caprilic acid	C8:0	0.2	5.2	-	0.2	6.2
Capric acid	C10:0	0.2	4.7	-	0.2	4.2
Lauric acid	C12:0	1.2	32.5	0.3	1.7	40.8
Miristic acid	C14:0	0.5	6.3	0.2	0.6	8.4
Palmitic acid	C16:0	10.0	5.9	19.4	15.6	6.9
Palmitoleic acid	C16:1 cis-9	0.5	-	3.5	3.5	-
Stearate acid	C18:0	8.7	6.5	-	1.6	2.4
Oleic acid	C18:1 cis-9	65.0	33.0	58.0	58.0	26.9
Linoleic acid	C18:2 cis-9,12	13.8	5.6	17.3	16.5	3.4
Linolenic acid	C18:3 cis-9,12,15	-	-	1.3	-	-

a Purchased from Cooperriacho Cooperative, located in the city of Mirabela, Minas Gerais, Brazil.

Density and kinematic viscosity were measured as a function of temperature, and the results are presented in Figure 3. The density values at 20 °C of approximately 0.91 g cm^–3^ and 0.92 g cm^–3^, align with those reported in the literature for oils with similar composition, and their evolution with temperature exhibits a linear behavior.

**Figure 3:**
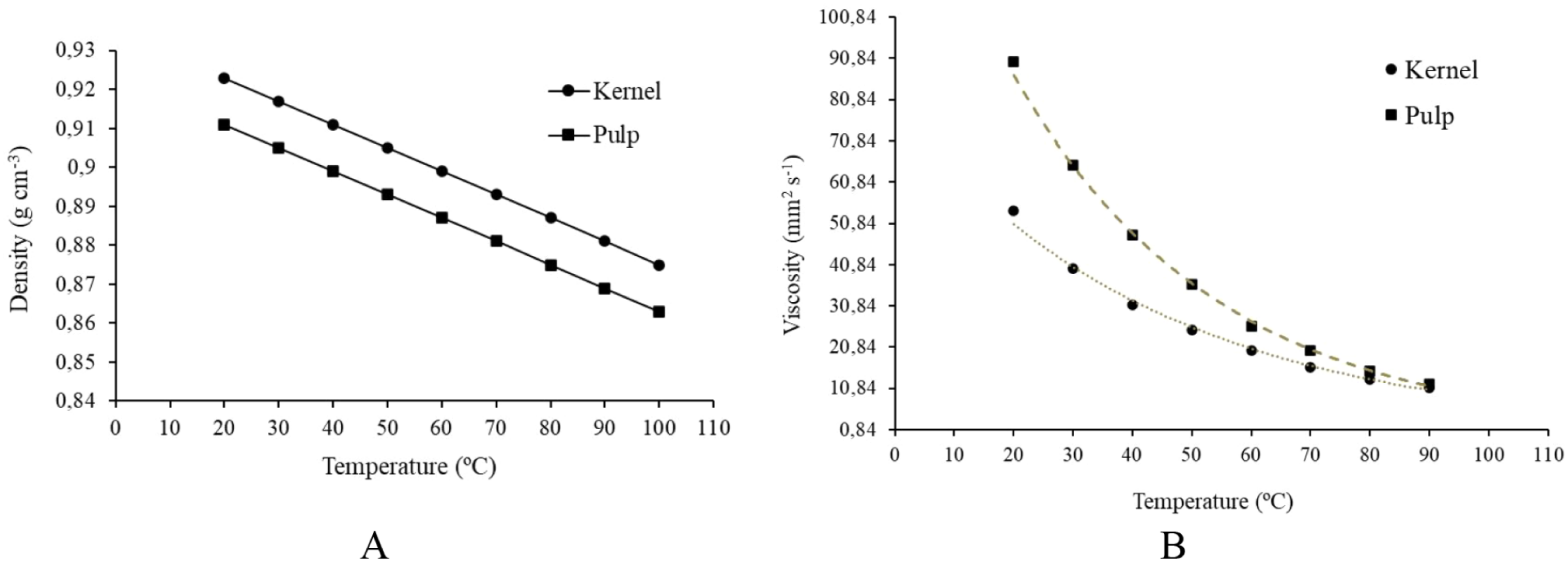
Density (A) and kinematic viscosity (B) of the macauba pulp and kernel oils.

At 40 °C, the pulp oil had a kinematic viscosity of 39 mm^2^ s^–1^, while the kernel oil showed a kinematic viscosity of 32 mm^2^ s^–1^, both consistent with their composition, and the values show an exponential trend with an increasing temperature. To interpret this tendency, equations were established considering the observed exponential behavior (eqs 2 and 3). Figure S1 (Supporting Information) presents both the behavior of the kinematic viscosity experimentally and the kinematic viscosity values predicted by the equations.

2 $$\mu_{kernel}=1.00\cdot e^{-0,029}+155.06$$

3 $$\mu_{pulp}=1.00\cdot e^{-0,023}+79.39$$

### 3.2 Biodiesel Production and Properties

Given the commercial interest in preserving Macauba kernel oil for higher-value applications, this study focuses on the use of pulp oil, which exhibits a composition more suitable for monounsaturated long-chain fatty acid methyl ester production—its mean—for biodiesel synthesis. This approach supports a more strategic and sustainable valorization of Macauba biomass, aligning energetic applications with market-driven priorities.

On the other hand, because we needed a large amount of Macauba pulp oil in order to obtain enough biofuel to perform physical-chemical properties studies of both biodiesel and its blends with S10 and S500 fossil diesel, as well as scale up its production in a continuous tubular reactor, we decided to afford Macauba pulp oil in the market instead of extracting it in our laboratory. The Macauba pulp oil was acquired from the Cooperriacho Cooperative, located in the city of Mirabela, Minas Gerais, Brazil, and the oil was extracted by cold mechanical pressing, followed by filtration to remove solid particles. Upon arrival at the laboratory, the oil was stored in plastic drums and kept frozen at −15 °C.

The commercial oil exhibits different physical-chemical properties, as well as fatty acid composition, than the one extracted in our laboratory. This was expected, as Macauba fruits were obtained after native palm trees grew in completely different edapho-climatic conditions. Indeed, this oil exhibited an acid value of 88.65 mg KOH g^–1^. The high acidity was probably a result of hydrolysis during extraction and storage conditions in the cooperative. The kinematic viscosity (35.4 mm^2^ s^–1^) and density (0.915 g cm^–3^) were at 40 and 25 °C, respectively. Note that the density was determined at this temperature because it was not completely liquid at 20 °C. These results, also different from those we obtained in our laboratory, are easily explained by differences in the composition of the oils (Table 3) and because of their high free fatty acid content.

Thus, due to the high acidity value (Table 3), biodiesel was produced from commercial pulp oil using a two-step process. For that, an initial esterification step using sulfuric acid as the catalyst was required. Following this, a base-catalyzed transesterification reaction was performed, resulting in a biodiesel product with an ester content greater than 98%. The physicochemical properties of the macauba biodiesel (MB), listed in Table 4, comply with the requirements established by the Brazilian National Agency for Petroleum, Natural Gas, and Biofuels (ANP).

**Table 4:** Physicochemical Properties of Macauba Oil, MMB, DS10, and DS500

Properties	Oil	MMB	DS10	DS500	Standard Method^a^
Viscosity (mm^2^ s^–1^)	35.4	4.4	2.65	2.72	ASTM D445
Acidity (mg KOH g^–1^)	88.65	1.05	-	-	AOCS CD 3d-63
Density (g m^–3^)	0.915^b^	0.879	0.842	0.838	NBR 7148
Heat of Combustion (MJ kg^–1^)	7560	40,940	46.8	46.9	ASTM D240

a Methods and specifications in accordance with specifications by the Brazilian National Petroleum Agency (ANP, 2025).

b Due to its low melting point, the density had to be determined at 25 °C to guarantee the homogeneity of the sample.

The fossil diesel samples used in this study (S10 and S500, hereafter referred to as DS10 and DS500) are commercially available in Brazil and are primarily differentiated by their maximum sulfur content, 10 and 500 mg kg^–1^ for DS10 and DS500, respectively. Selected physicochemical properties of these diesel samples are also presented in Table 4, and all values fall within the specifications outlined in Brazil (RESOLUÇÃO ANP N° 968, DE 30 DE ABRIL DE 2024).

It is important to note that MMB synthesized in this study, like biodiesel derived from other oilseeds, exhibits density and kinematic viscosity values that are, to a certain extent, compatible with the use in compression-ignition engines. These properties are essential to ensure proper atomization and combustion behavior when the fuel is injected into the engine chamber.

Blends containing 5% to 95% v/v of MMB were prepared using DS10 and DS500, and for each one, density, kinematic viscosity, and calorific value were determined. The major objective was to monitor the evolution of these properties across different biodiesel/diesel ratios, aiming to identify the maximum biodiesel content compatible with regulatory limits. Additionally, the influence of the temperature on these properties was investigated. Figure 4 presents the prepared blends, revealing distinct color variations; formulations with MMB+DS10 exhibit a yellowish tone, while those with MMB+DS500 display a reddish appearance.

**Figure 4:**
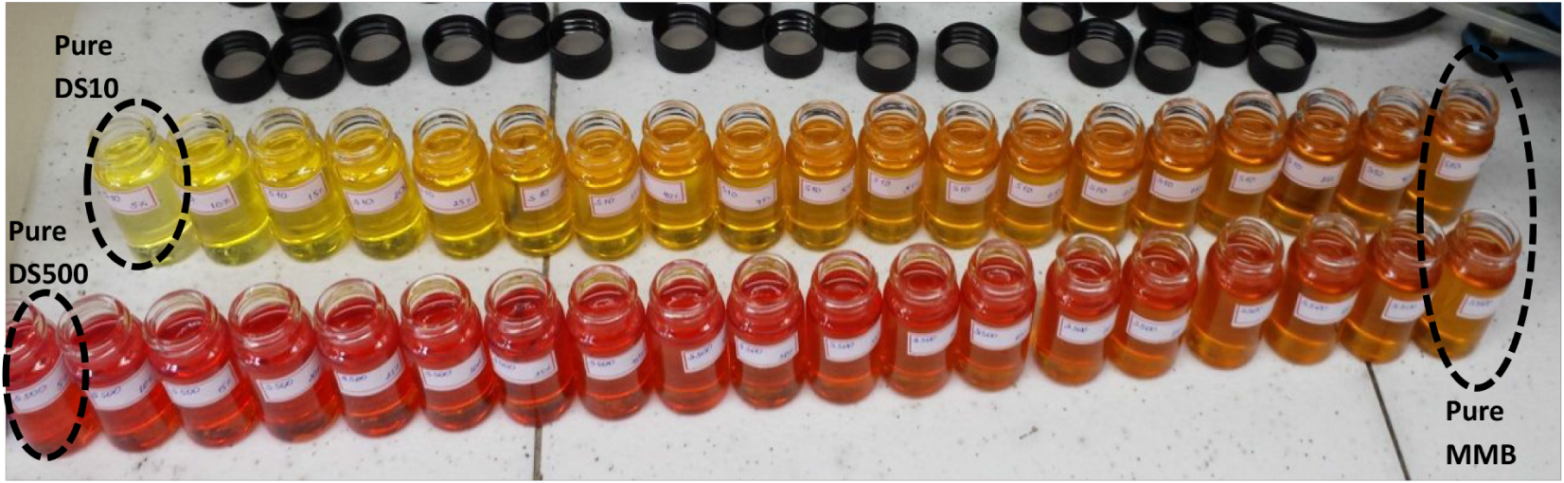
Color aspect of MMB, DS10, and DS500 blends.

The measured density values of the mixtures between MMB and fossil diesel DS10 and DS500 are presented in Table T1 (Supporting Information), and Figure 5 illustrates the evolution of these results across the different blend ratios. As previously mentioned, MMB exhibits a higher density compared to the fossil diesel samples, and as the biodiesel content in the blends increases, a gradual rise in density is observed, as expected.

**Figure 5:**
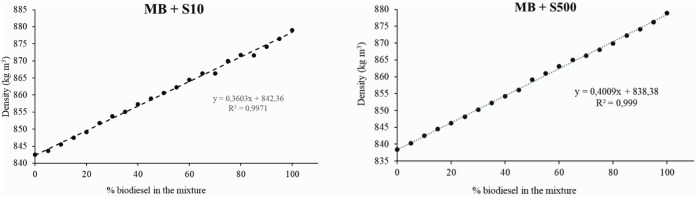
Density profile of MMB blended with fossil diesel (DS10 and DS500).

In both cases (Figure 5), a linear relationship between the density and biodiesel content can be observed. The Kay mixing rule model (eq 4) can be successfully applied, allowing the prediction of density values for various blends at a fixed temperature (ρ_b_ = density of the blend (kg·m^–3^); *V*_1_ = biodiesel fraction in the blend; ρ_1_ = density of biodiesel (kg·m^–3^); *V*_2_ = diesel fraction in the blend; ρ_2_ = density of diesel (kg·m^–3^)).^20,21^

4 $$\rho_{b}=V_{1}\times \rho_{1}+V_{2}\times \rho_{2}$$

For both MMB+DS10 and MMB+DS500 blends, good agreement was observed between the experimental and predicted density values, with correlation coefficients (*r* ^2^) of 0.9971 and 0.9990, respectively, based on eq 4. These results are consistent with the model proposed in literature.^20^

To assess the behavior of density as a function of temperature—an important factor given that the fuel is marketed throughout the countries, which can present significant climatic variations—an additional study was carried out at different temperatures, in addition to the reference temperature of 20 °C specified by the ASTM D4052-11 standard (Figure S2, Supporting Information).

Furthermore, a linear relationship between density and temperature was observed within the studied range, as confirmed by the *R* ^2^ values obtained (Figure S3, Supporting Information). Nonetheless, the evolution of density as a function of temperature is inversely proportional, a trend also observed in the literature in the studies of the relationship between density and temperature for blends of used cooking oil biodiesel with diesel over a temperature range from 0 to 130 °C.^22^

Another important parameter in evaluating the use of biodiesel and its blends with diesel is the kinematic viscosity, as it measures the internal resistance to fluid flow and is a critical factor in the combustion process within the engine chamber. High viscosity can result in nonuniform combustion, leading to the deposition of residues on the internal components of the engine.^23^

Table T2 (Supporting Information) presents the kinematic viscosity results for MMB and its blends with DS10 and DS500 diesel. Figure 6 illustrates the behavior of the experimentally determined kinematic viscosity as a function of the biodiesel content in the blends, as well as the predicted viscosity values based on the exponential model.

**Figure 6:**
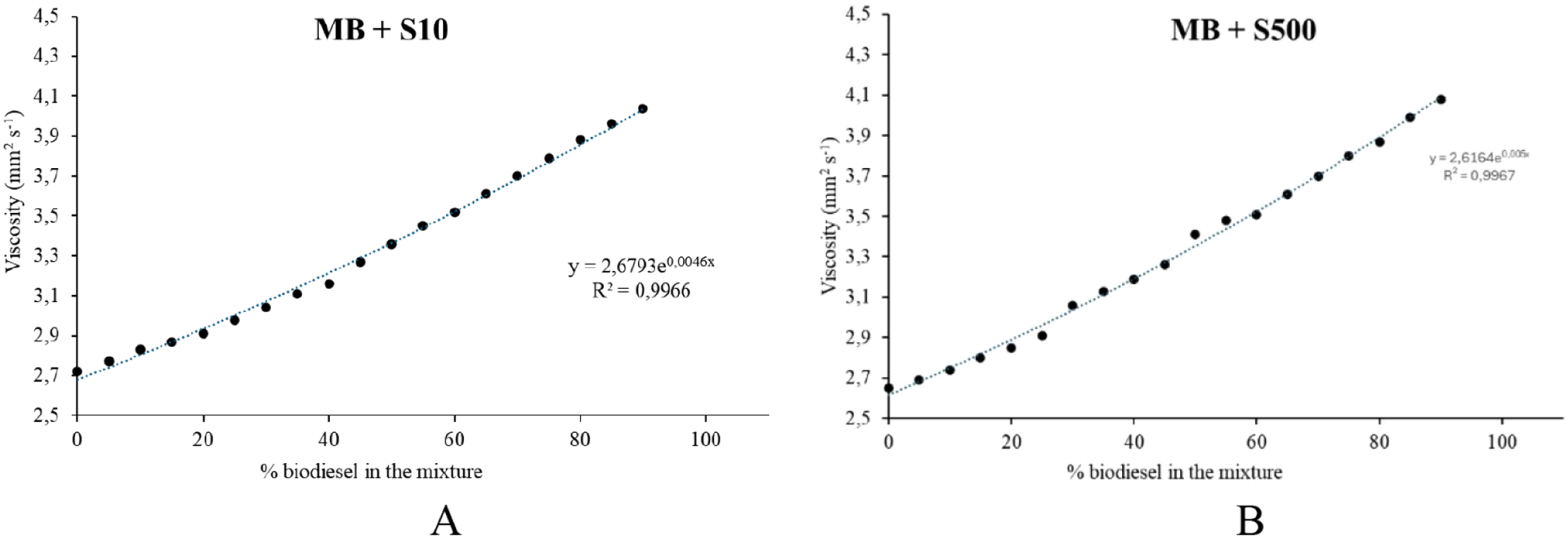
Kinematic viscosity profile of MMB blended with fossil diesel (DS10 and DS500).

Similarly to the density evaluation, a study of the kinematic viscosity behavior at different temperatures was conducted, in addition to the standard temperature of 40 °C established by ASTM D445-12. The kinematic viscosity behavior across these temperatures is presented in Figure S3.

Figure S3 (Supporting Information) likewise shows an exponential relationship between kinematic viscosity and temperature, which can be confirmed by the *r* ^2^ values obtained. The analysis of the results indicates that kinematic viscosity decreases with increasing temperature, following an exponential Arrhenius-type dependence, described by eq 4 (υ is the kinematic viscosity; υ_0_ = viscosity at high temperature; A = the parameter determining the curvature of the fit; T = absolute temperature (Kelvin)).

5 $${\upsilon =\upsilon }_{0}e^{A/T}$$

The heating value is defined as the amount of energy released as heat by combustion of a unit mass of fuel. It is a key property, as the maximum power output of an engine during operation depends on the heating value of the fuel.^24^ Table T3 presents the heating value results for MMB and its blends with DS10 and DS500 diesel. Figure 7 illustrates the behavior of the experimentally determined heating value as a function of the biodiesel content in the blends.

**Figure 7:**
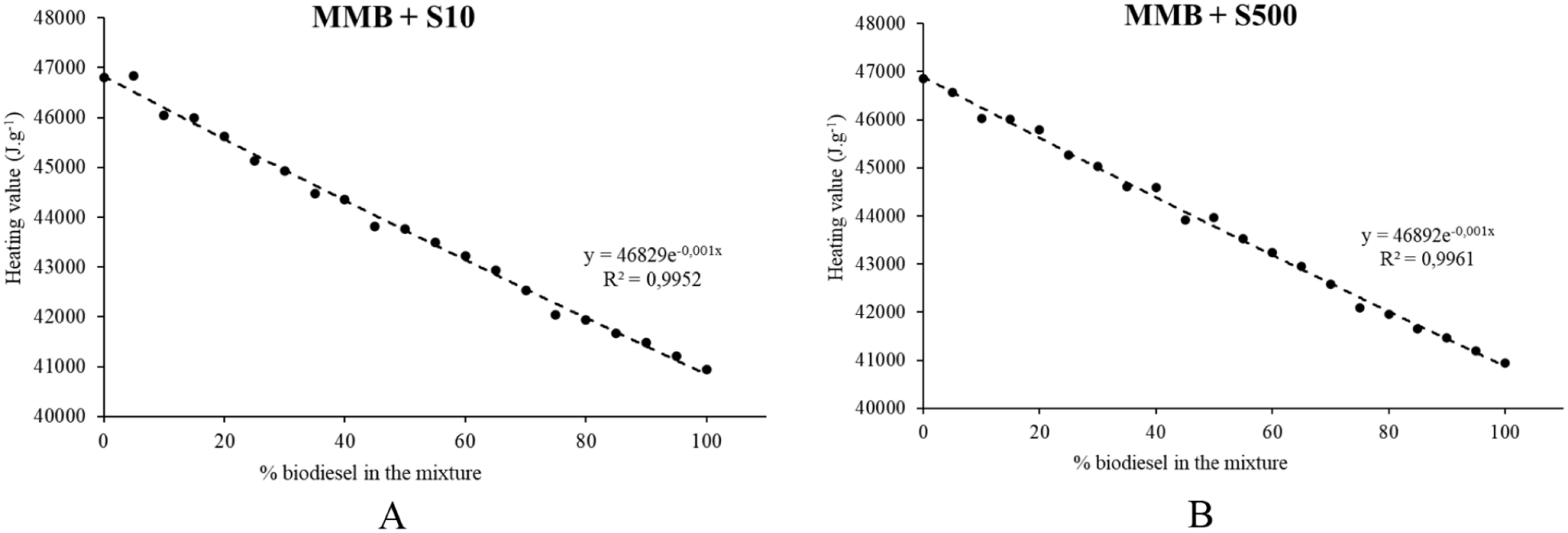
Heating value profile of MMB blended with fossil diesel (DS10 and DS500).

As shown in Figure 7, the heating value of the blends decreases linearly with increasing the biodiesel content. This behavior is consistent with the intrinsic characteristics of biodiesel, which typically presents lower heating values compared to petroleum-derived diesel due to its higher oxygen content. Despite this slight reduction, the heating value obtained for the blends remains within acceptable limits for use in conventional diesel engines.

In Brazil, the specifications governing biodiesel–diesel blends are established by the ANP, as outlined in Resolution No. 909 of December 5, 2022, to ensure fuel quality and optimal engine performance. With respect to density, the regulatory limits for diesel–biodiesel blends are set between 0.815 and 0.850 g cm^–3^ for S10 diesel and between 0.815 and 0.865 g cm^–3^ for S500 diesel. Concerning kinematic viscosity at 40 °C, blends must comply with the specifications defined for biodiesel, specifically within the range of 2.0 to 4.5 mm^2^ s^–1^, regardless of the type of fossil diesel employed. Based on these regulatory parameters, it is feasible to incorporate up to 60% (v/v) of MMB into S10 diesel while maintaining compliance with ANP standards. In contrast, blending MMB with S500 diesel would allow for incorporation levels of up to 90% (v/v) without exceeding the prescribed limits.

Moreover, although not explicitly regulated, the lower heating value of MMB is approximately 40,940 J kg^–1^, representing a reduction of about 13% compared to conventional fossil diesel, which exhibits a value of approximately 46,800 J kg^–1^. Consequently, increasing the proportion of biodiesel in the blend leads to a decrease in the overall energy content, an aspect that must be considered in engine performance evaluations. Nevertheless, under the conditions assessed, the reduction in the released combustion energy would not be expected to cause significant losses in engine power output.

### 3.3 Evaluation of Biodiesel Production from Macauba Oil in a Continuous Tubular Reactor

Although initial experiments were conducted at pilot scale as described in the previous session, advancing toward continuous reactor systems for macauba biodiesel production is strategically significant. Continuous processes offer notable advantages over batch operations, such as enhanced heat and mass transfer, improved reaction control, and reduced production costs, facilitating the scalability to industrial levels. Studies have demonstrated that continuous reactors can achieve higher efficiency and consistent product quality in biodiesel production.^25–27^

Specifically, the high oil yield and adaptability of macauba, coupled with the operational benefits of continuous processing, underscore its viability for large-scale biodiesel production. To validate these theoretical- and literature-based advantages, experimental trials were essential to assess the practical performance of macauba oil transesterification under continuous flow conditions. Despite the promising characteristics of continuous reactors (such as enhanced reaction control and scalability), the specific behavior of macauba oil in such systems had not yet been thoroughly explored. Therefore, experimental tests were designed to investigate critical parameters like conversion efficiency, operational stability, and product quality, providing concrete data to substantiate the process’s viability. This experimental approach aimed to bridge the gap between conceptual benefits and real-world applications, ensuring that the continuous production of macauba biodiesel meets both technical and regulatory requirements.

The operation of the continuous tubular reactor is described in detail in Section 2.8. The reaction products were collected for analysis only after the reactor reached steady-state conditions, ensuring representativeness of the data obtained. Table 5 presents the average biodiesel yields achieved under steady-state operation using unrefined macauba oil as the feedstock for each catalyst bed in the presence of methanol.

**Table 5:** Sequence and Conditions of the Transesterification Reactions Performed on the Continuous Prototype with Each Catalyst Bed^a^

Reaction	T_reator_ (°C)	Methanol and oil ratio (mol mol^–1^)	Reactor residence time (h)	(Al_2_O_3_)_4_(ZnO) Average yield (%)	(Al_2_O_3_)_4_(SnO) yield (%)
1	140	6	4	55 ± 4	50 ± 3
2	140	6	6	60 ± 5	54 ± 5
3	140	9	4	62 ± 3	61 ± 2
4	140	9	6	65 ± 4	64 ± 5
5	150	6	4	65 ± 5	66 ± 4
6	150	6	6	70 ± 4	71 ± 5
7	150	9	4	75 ± 3	73 ± 6

a Preparation described in Section 2.8.

Both catalysts exhibited similar biodiesel yields, as expected, since batch bench-scale reactions have also shown comparable results.^16,17,27^ Higher yield values could likely be achieved by increasing the residence time and reactor temperature and/or performing consecutive reactions. Indeed, because esterification is the main reaction taking place, removing water and performing a consecutive reaction has been shown to be effective to achieve high yield in methyl-fatty acid esters using Macauba and cadmium oxide as catalysts in a batch reactor.^28^

The experimental results presented herein align with the advantages of continuous processing, confirming the technical feasibility of macauba oil transesterification in a continuous tubular reactor. The data reveal consistent biodiesel yields across varying operational conditions, demonstrating the system’s robustness and reproducibility. Moreover, the observed conversion rates and product quality indicators, such as ester content, viscosity, and density, are in accordance with established fuel standards, reinforcing the practical applicability of continuous reactors for macauba biodiesel production. These findings not only corroborate with the literature but also provide empirical evidence supporting the scalability of this process to industrial dimensions.

### 3.4 Pyrolysis of Macauba Lignocellulosic Biomass

Lignocellulosic material from macauba fruit was pyrolyzed under a nitrogen atmosphere to obtain biochar at different temperatures. As shown in Table 6, the temperature plays a key role in determining biochar yield. As the pyrolysis temperature increased, the biochar yield decreased, which is attributed to the loss of volatile compounds and the progressive densification of carbon–carbon bonding, increasing the formation of ordered carbonaceous structures.

**Table 6:** Biochar Yields at Different Pyrolysis Temperatures for Biochar with Extractives and Unextracted Biochar

	Temperature (°C)	Yield^a^ (wt %)
Biochar with extractives	400	41.10 ± 1.17%
	500	21.62 ± 0.75%
	600	19.85 ± 0.39%
Unextracted biochar	400	37.56 ± 1.74%
	500	26.75 ± 1.06%
	600	24.40 ± 0.69%

a Yield (wt %) determined gravimetrically.

The carbon, hydrogen, nitrogen, and oxygen contents of the biochars are listed in Table 7. Elemental analysis shows that, compared to the original biomass, the resulting biochars exhibit a decrease in oxygenated groups, an increase in nitrogen content, and, most notably, a higher carbon concentration. These changes indicate the successful formation of biochar and the loss of volatile compounds during pyrolysis. The main difference in elemental composition between biochar derived from biomass with extractives and that without extractives (Table 7) lies in the carbon content. The extractive-free biomass produced biochar with a lower carbon content due to the prior removal of oil, which results in the loss of hydrocarbon chains and, consequently, lower carbon and hydrogen contents. The H/C and O/C atomic ratios in both cases show the same trend with increasing pyrolysis temperature, which highlights the enhanced energy potential of the biochars and reflects the transformation of oxygen-containing compounds present in lignocellulosic materials.

**Table 7:** Elemental Analysis (%), Higher Heating Value (HHV), and Ash Content (%) for Biochar with Extractives and Unextracted Biochar

	T (°C)	C (%)	H (%)	N (%)	O (%)	O/C (%)	H/C (%)	HHV (kJ/kg)	Ash (%)
Biochar with extractives	400	58.09	5.43	0.80	33.05	0.4273	1.1215	23.25	2.63
	500	60.34	3.64	0.84	32.32	0.4023	0.7238	22.90	2.86
	600	65.55	2.48	0.93	27.95	0.3203	0.4539	22.00	3.1
Unextracted biochar	400	55.37	5.37	0.12	36.71	0.4980	1.1636	17.69	2.43
	500	57.12	3.99	1.18	35.05	0.4609	0.8381	21.00	2.66
	600	58.76	1.75	0.08	36.52	0.4668	0.3573	23.12	2.89

Therefore, lower carbon and hydrogen contents were observed for the macauba samples containing extractives. These results are consistent with those reported in the literature,^29,30^ which described an H/C ratio higher than that of the standard biomass region in the Van Krevelen diagram (Figure 8). After pyrolysis, each lignocellulosic component underwent different reaction mechanisms, such as decarboxylation, dehydration, and demethylation, resulting in the formation of pyrolysis products such as biochar, bio-oil, and syngas. Consequently, the composition of the raw material shifted toward a more carbon-rich profile, with reduced polarity (O/C) and increased aromaticity (lower H/C), as the severity of the pyrolysis intensified (Table 7).

**Figure 8:**
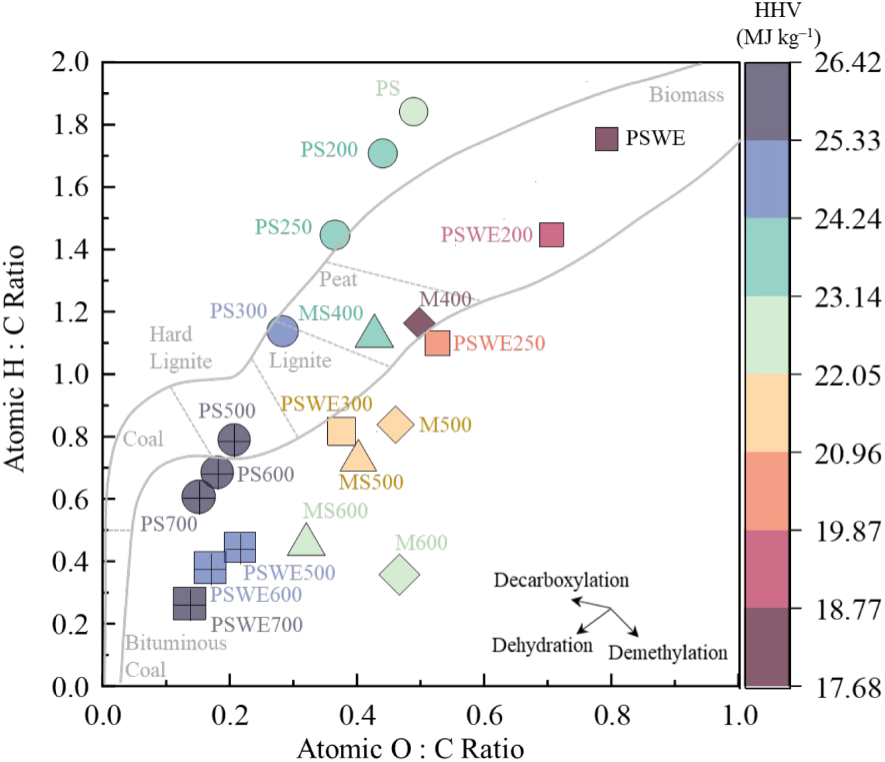
Van Krevelen diagram for macauba biochar with extractives (M400, M500, and M600) and macauba unextracted biochar (MS400, MS500, and MS600 C).

The biochar obtained from macauba biomass containing extractives showed a greater decrease in both the O/C and H/C ratios, which is attributed to the degradation of extractive compounds. As observed, the extractive-free samples exhibited even greater reductions in H/C and O/C ratios; however, only the biochars produced at 400 °C—both with and without extractives—remained within the recommended limits (H/C = 0.6 and O/C = 0.4, respectively) established by the European Biochar Certificate for biochar materials.

The higher heating value (HHV) is influenced by these atomic ratios, and the biochar’s energy density increases as the H/C and O/C ratios decrease with increasing pyrolysis temperature.^30^ Nevertheless, for the biochars produced from extractive-containing macauba biomass, a higher HHV was observed. In contrast, the extractive-free samples exhibited an inverse trend, likely due to greater structural packing, which corresponds to a lower degree of graphitization.

As shown in Table 8, the materials containing extractives presented a higher graphitization index, indicating lower graphitization; that is, their carbonaceous structures are more disordered. Temperature also influenced the graphitization degree, with the biochar derived from extractive-containing biomass pyrolyzed at 400 °C exhibiting the lowest degree of graphitization.

**Table 8:** Graphitization Index of Macauba Biochars

	Temperature (°C)	Graphitization index
Biochar with extractives	400	1.25
	500	0.77
	600	0.37
Unextracted biochar	400	0.29
	500	0.26
	600	0.28

Analysis of mass loss in both types of biochar (with and without extractives) revealed that increasing the temperature led to greater mass loss. Biochars produced at 600 °C exhibited minimal degradation (average mass loss of ∼5%), as most thermal decomposition had already occurred during pyrolysis. When extractive-free macauba biochar was compared with that derived from biomass containing extractives, a higher mass loss was observed in the latter. This is attributed to the decomposition of oil components that remained in the biomass and were not fully degraded during the pyrolysis process. For samples prepared at the same temperature, the variation in mass was consistently greater in the biochar with extractives. It was also observed that the surface areas of all biochars were very low, less than 1 m^2^ g^–1^.

## 4 Conclusion

This study highlights the potential of macauba as a multifaceted feedstock for biofuel and biochar production. The physicochemical characterization of pulp and kernel oils confirmed their distinct fatty acid profiles and respective industrial applications. Biodiesel synthesized from commercial macauba pulp oil exhibited properties compliant with ANP standards, and blending tests demonstrated compatibility with fossil diesel up to high volumetric fractions. Furthermore, biodiesel production in a continuous tubular reactor was shown to be technically viable, achieving promising yields. The valorization of lignocellulosic residues through pyrolysis resulted in biochars with favorable elemental composition and heating values, particularly from biomass containing extractives. These findings support the integration of macauba into sustainable biorefinery strategies that encompass both energy and material recovery, contributing to the diversification of renewable energy sources and the development of low-carbon technologies. However, the main limitation of this study is that all evaluations were performed under laboratory conditions, which do not fully represent the large-scale variability in macauba biomass or processing performance. Future studies should investigate the influence of edaphoclimatic cultivation conditions on both oils and lignocellulosic biomass productivity and characteristics, as well as assess biodiesel and biochar production at pilot scale to validate the integrated biorefinery approach and economic feasibility.

## Supplementary Material



## Data Availability

All relevant data are available within the article and its Supporting Information.

## Notes

Density results (Table T1), viscosity results (Table T2), and HHV results (Table T3) of MMB+DS10 and MMB+DS500 blends; behaviour of the kinematic viscosity (Figure S1); density as a function of temperature (Figure S2); viscosity as a function of temperature (Figure S3) (PDF)
