# Supplementary material for: Macauba (Acrocomia aculeata) as a Bioenergy Platform: Integrated Biodiesel Production and Lignocellulosic Biomass Valorization
Source: ACS Omega. 2025 Dec 12;10(51):63608–19. doi: 10.1021/acsomega.5c10973 (PMC12756744; doi:10.1021/acsomega.5c10973)

Macauba (*Acrocomia aculeata*) as a Bioenergy Platform: Integrated Biodiesel Production and Lignocellulosic Biomass Valorization

Daniel Chagas Nascimento[a], Ewerton Henrique de Souza Santos[b], Fábio Moreira da Silva[a], Diego Coelho Barroso dos Santos[a], Edgar Amaral Silveira[c], Janaína Heberle Bortoluzzi[a], Grace Ferreira Ghesti[a], Mario Roberto Meneghetti[a], Paulo Anselmo Ziani Suarez[b,*], Simoni Margareti Plentz Meneghetti[a,*]

[a] Laboratory of Bioprocess, Materials and Fuels – Institute of Chemistry, University of Brasília, P.O. Box 4478, 70919-970 Brasília, DF, Brazil, e-mail: psuarez@unb.br

[b] Group of Catalysis and Chemical Reactivity– Institute of Chemistry and Biotechnology – Federal University of Alagoas, 57072-000, Maceió, AL, Brasil, e-mail: simoni.plentz@gmail.com

[c] Mechanical Sciences Graduate Program, Laboratory of Energy and Environment, University of Brasília, 70919-970 Brasília, DF, Brazil

Supplementary information

Table T1. Density results of mixtures MMB+DS10 and MMB+DS500

	MMB+DS10		MMB+DS500	
MMB amount (%)	Density (kg.m^{-3})		Density (kg.m^{-3})	
0	842.5	0.1	838.5	0.1
5	843.6	0.3	840.2	0.1
10	845.5	0.1	842.5	0.1
15	847.4	0.1	844.5	0.1
20	849.1	0.1	846.3	0.1
25	851.8	0.1	848.1	0.1
30	853.7	0.1	850.2	0.1
35	855.1	0.2	852.2	0.1
40	857.3	0.2	854.2	0.2
45	859.0	0.2	856.1	0.1
50	860.6	0.1	859.1	0.1
55	862.3	0.1	861.0	0.1
60	864.5	0.2	863.2	0.1
65	866.4	0.3	865.0	0.1
70	866.3	0.2	866.2	0.1

75	870.0	0.1		868.1	0.1
80	871.7	0.2		869.9	0.1
85	871.6	0.0		872.3	0.1
90	874.2	0.1		874.1	0.1
95	876.5	0.1		876.2	0.1
100	879.0	0.2		879.0	0.2

The values presented are the average of triplicate measurements.

Table T2. Viscosity results of blends MMB+DS10 and MMB+DS500

	MMB+DS10			MMB+DS500	
MMB amount (%)	Viscosity ($mm^2.s^{-1}$)			Viscosity ($mm^2.s^{-1}$)	
0	2.65	0.007		2.72	0.019
5	2.69	0.001		2.77	0.003
10	2.74	0.003		2.83	0.002
15	2.80	0.006		2.87	0.004
20	2.85	0.006		2.91	0.002
25	2.91	0.000*		2.98	0.002
30	3.06	0.001		3.04	0.001
35	3.13	0.000*		3.11	0.006
40	3.19	0.002		3.16	0.000*
45	3.26	0.001		3.27	0.000*
50	3.41	0.003		3.36	0.000*
55	3.48	0.002		3.45	0.001
60	3.51	0.001		3.52	0.001
65	3.61	0.002		3.61	0.001
70	3.70	0.001		3.70	0.001
75	3.80	0.001		3.79	0.001
80	3.87	0.007		3.88	0.001
85	3.99	0.009		3.96	0.000*
90	4.08	0.005		4.04	0.003
95	4.22	0.001		4.22	0.002
100	4.41	0.003		4.41	0.003

The values presented are the average of triplicate measurements.

Table T3. Higher heating value (HHV) results of blends MMB+DS10 and MMB+DS500

	MMB+DS10		MMB+DS500	
MMB amount (%)	HHV ($J.g^{-1}$)		HHV ($J.g^{-1}$)	
0	46810	0.57	46862	0.11
5	46831	1.43	46568	0.19
10	46045	0.17	46034	0.20
15	46001	0.13	46015	0.11
20	45629	0.25	45787	0.42
25	45136	0.01	45260	0.44
30	44927	0.05	45036	0.04
35	44475	0.06	44604	0.40
40	44351	0.16	44597	0.03
45	43809	0.03	43910	0.26
50	43766	0.18	43970	0.29
55	43501	0.10	43526	0.08
60	43221	0.56	43240	0.13
65	42929	0.40	42948	0.17
70	42534	0.00	42580	0.07
75	42040	0.06	42089	0.20
80	41945	0.01	41964	0.22
85	41670	0.05	41648	0.16
90	41482	0.02	41466	0.12
95	41216	0.04	41195	0.02
100	40940	0.17	40940	0.03

The values presented are the average of triplicate measurements.

Fig. S1. Behavior of the kinematic viscosity: experimental and predicted data.

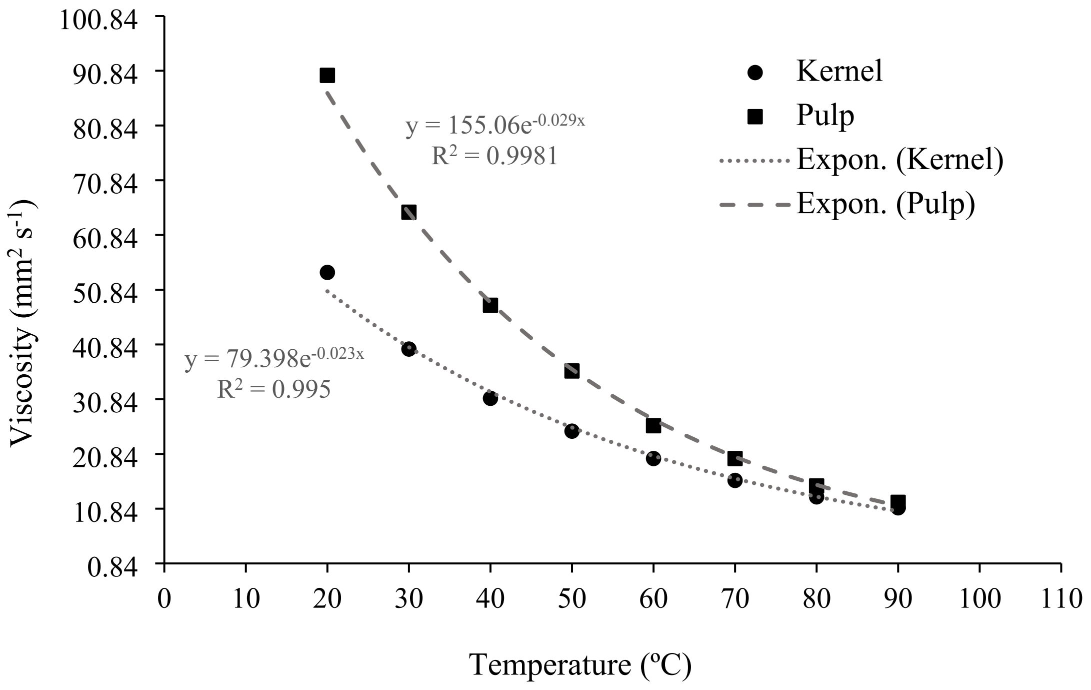

Fig. S2. Density as a function of temperature.

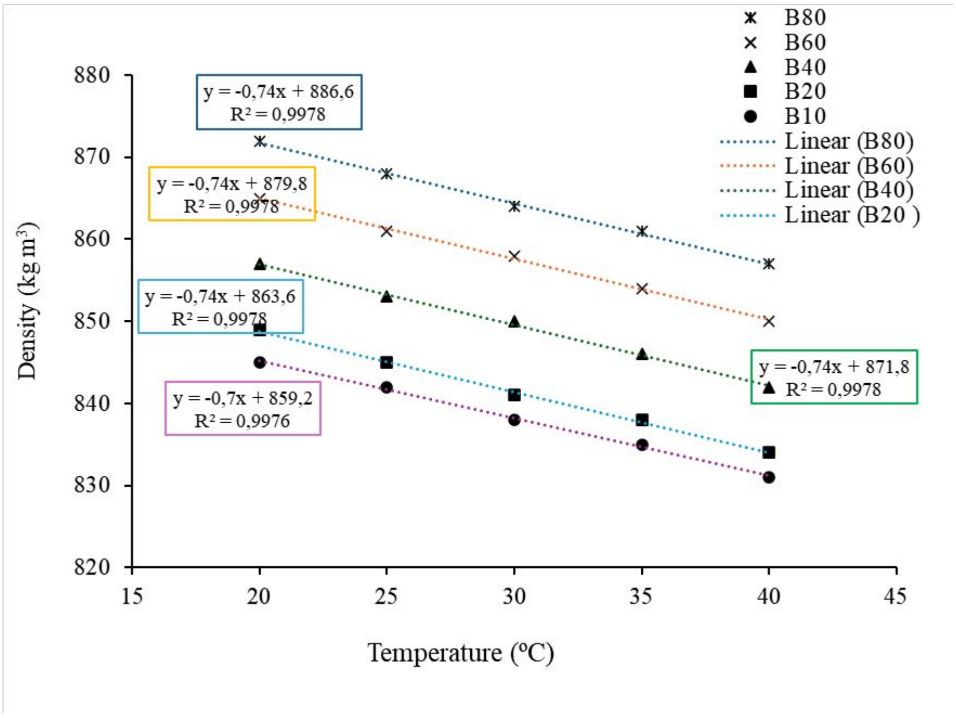

Fig. S3. Viscosity as a function of temperature.

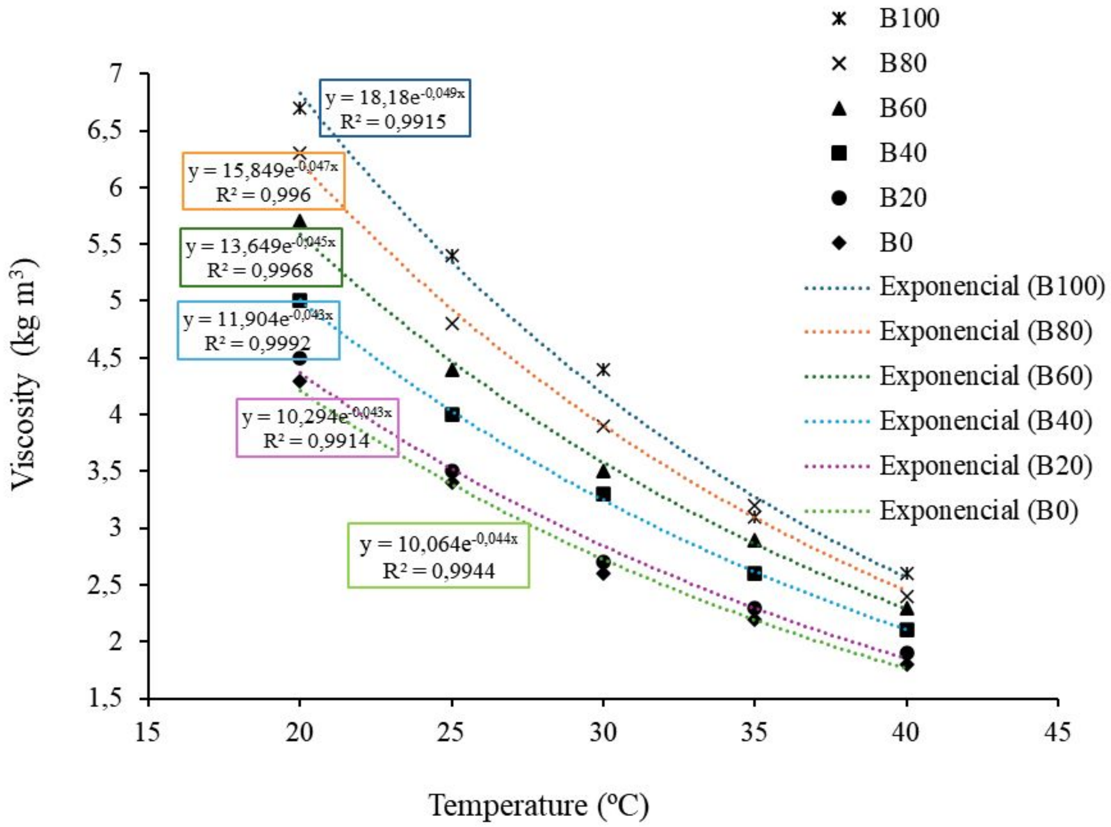

Supplement: Supplementary file 1 [file ao5c10973_si_001.pdf]
